# Soil Chemical Properties Barely Perturb the Abundance of Entomopathogenic *Fusarium oxysporum*: A Case Study Using a Generalized Linear Mixed Model for Microbial Pathogen Occurrence Count Data

**DOI:** 10.3390/pathogens7040089

**Published:** 2018-11-16

**Authors:** Lav Sharma, Irene Oliveira, Fernando Raimundo, Laura Torres, Guilhermina Marques

**Affiliations:** 1 CITAB—Centre for the Research and Technology of Agro-Environmental and Biological Sciences, University of Trás-os-Montes and Alto Douro, UTAD, 5001-801 Vila Real, Portugal; ioliveir@utad.pt (I.O.); fraimund@utad.pt (F.R.); ltorres@utad.pt (L.T.); gmarques@utad.pt (G.M.); 2 CEMAT-IST-UL—Centre for Computational and Stochastic Mathematics, University of Lisbon, 1649-004 Lisbon, Portugal

**Keywords:** entomopathogenic fungi, soil chemistry, microbial ecology, vineyards, *Fusarium oxysporum*, generalized linear mixed model

## Abstract

*Fusarium oxysporum* exhibits insect pathogenicity—however, generalized concerns of releasing phytopathogens within agroecosystems marred its entomopathogenicity-related investigations. In a previous study, soils were sampled from Douro vineyards and adjacent hedgerows. In this study, 80 of those soils were analyzed for their chemical properties and were subsequently co-related with the abundance of entomopathogenic *F. oxysporum*, after insect baiting of soils with *Galleria mellonella* and *Tenebrio molitor* larvae. The soil chemical properties studied were organic matter content; total organic carbon; total nitrogen; available potassium; available phosphorus; exchangeable cations, such as K^+^, Na^+^, Ca^2+^, and Mg^2+^; pH; total acidity; degree of base saturation; and effective cation exchange capacity. Entomopathogenic *F. oxysporum* was found in 48 soils, i.e., 60% ± 5.47%, of the total soil samples. Out of the 1280 insect larvae used, 93, i.e., 7.26% ± 0.72%, were found dead by entomopathogenic *F. oxysporum*. Stepwise deletion of non-significant variables using a generalized linear model was followed by a generalized linear mixed model (GLMM). A higher C:N (logarithmized) (*p* < 0.001) and lower exchangeable K^+^ (logarithmized) (*p* = 0.008) were found significant for higher fungal abundance. Overall, this study suggests that entomopathogenic *F. oxysporum* is robust with regard to agricultural changes, and GLMM is a useful statistical tool for count data in ecology.

## 1. Introduction

Entomopathogenic fungi are the natural biological control agents of insect pests [1]. The fungi belonging to *Fusarium* Link ex Grey (Hypocreales: Nectriaceae) are widely known as plant pathogens and saprophytes. Among animals, *Fusarium* spp. are quite abundantly associated with insects from different orders, i.e., Coleoptera, Diptera, Hemiptera, Isoptera, Lepidoptera, and Orthoptera [2,3]. A previous study emphasized the use of different *Fusarium* spp. as biological control agents for the agricultural insect pests, and aroused concern towards the limited research in this direction, pertaining to a generalized apprehension of releasing phytopathogens and related toxins in the environment [4]. The commonly occurring fusaria from insects were termed “insecticolous fungi” [4]. Another study suggested that *Fusarium* spp., which can kill insects, can be good candidates for insect biological control in agroecosystems. Because these fusaria sometimes demonstrate high host specificity, can be easily cultured in a laboratory setting, can survive in fields as facultative pathogens, and are not all harmful to plants [5].

*Fusarium oxysporum* Schlechtendahl is a widely known pathogen of plants and animals, including infections in humans; however, safe fusaria also exist in nature. Previous studies provide compelling reasons to consider fungi like *F. oxysporum* for biological control of insect pests [4,5]. Another recent study presented a detailed dose-response curve and histological evidence of *F. oxysporum* infections, and subsequent mortalities, in the larvae of wax moth *Galleria mellonella* Linnaeus (Pyralidae: Lepidoptera) [6]. Therefore, it was a proof-of-concept study demonstrating the entomopathogenicity of *F. oxysporum*.

Soil is an excellent reservoir of insect-pathogenic fungi (IPF). However, only a few studies report the effects of physicochemical properties of soil on the distribution of IPF. Previous studies in this direction primarily focused on IPF *Beauveria bassiana* (Balsamo) Vuillemin (Hypocreales: Cordycipitaceae), and *Metarhizium anisopliae* (Metschnikoff) (Hypocreales: Clavicipitaceae) [7,8,9,10,11,12]. According to our knowledge, to date there is no report that focuses on soil chemical properties in terms of the abundance of IPF *F. oxysporum*.

In this study, authors analyzed the chemical properties of the soils, including percentage organic matter content (OM); total organic carbon (C); total nitrogen (N); available potassium (K); available phosphorus (P); exchangeable ions such as potassium (K^+^), sodium (Na^+^), calcium (Ca^2+^) and magnesium (Mg^2+^); pH in H_2_O; total acidity (TA); degree of base saturation (DBS); and effective cation exchange capacity (ECEC), and investigated their effects on the natural abundance of entomopathogenic *F. oxysporum*. To enhance variations in the chemical properties, different soils were considered. Soils were (a) different in texture—i.e., medium-texture (more balanced mixture of sand, silt, and clay) or coarse-texture (high proportion of sand)—(b) sampled from varying habitat types—i.e., cultivated vineyards or adjacent hedgerows, mainly constituted of oak (*Quercus* spp. Linnaeus, Fagaceae) and pine trees (*Pinus* spp. Linnaeus, Pinaceae)—and (c) either treated with herbicides or left untreated.

Statistical modeling has been useful in entomology and related studies [13,14]. The generalized linear mixed model (GLMM) is a widely known tool in ecology for count data [15]. In terms of IPF, count data is of great relevance; however, investigations implementing GLMM to study IPF occurrences are limited [16].

The objectives of this work were (a) to understand the effects of soil chemical properties on IPF *F. oxysporum* occurrences in the soil, and (b) to demonstrate the usefulness of GLMM in studying the abundance of microbial pathogens—for example, IPF. This study goes a step further and provides a newer prospective among the ongoing efforts to utilize microbial entomopathogenicity in insect pest biological control within agroecosystems. To the best of our knowledge, this is the first report on the effects of the soil chemical properties with respect to inter-kingdom host pathogen *F. oxysporum*.

## 2. Results

### 2.1. Infection Frequencies

A total of 80 soils were selected to access the occurrence of entomopathogenic *F. oxysporum*, in terms of their chemical properties (Table 1). Out of these, 48 samples were found positive for the fungus (i.e., 60% ± 5.47%). A total of 93 *F. oxysporum* strains were isolated and found to be insect-pathogenic, after testing 1280 insect larvae in total—16 larvae in each of the 80 soils. The frequency of infection among baited larvae by *F. oxysporum* was 7.26% ± 0.72%.

### 2.2. Effect of Soil Chemical Properties on Fungal Abundance

**Figure 1:**
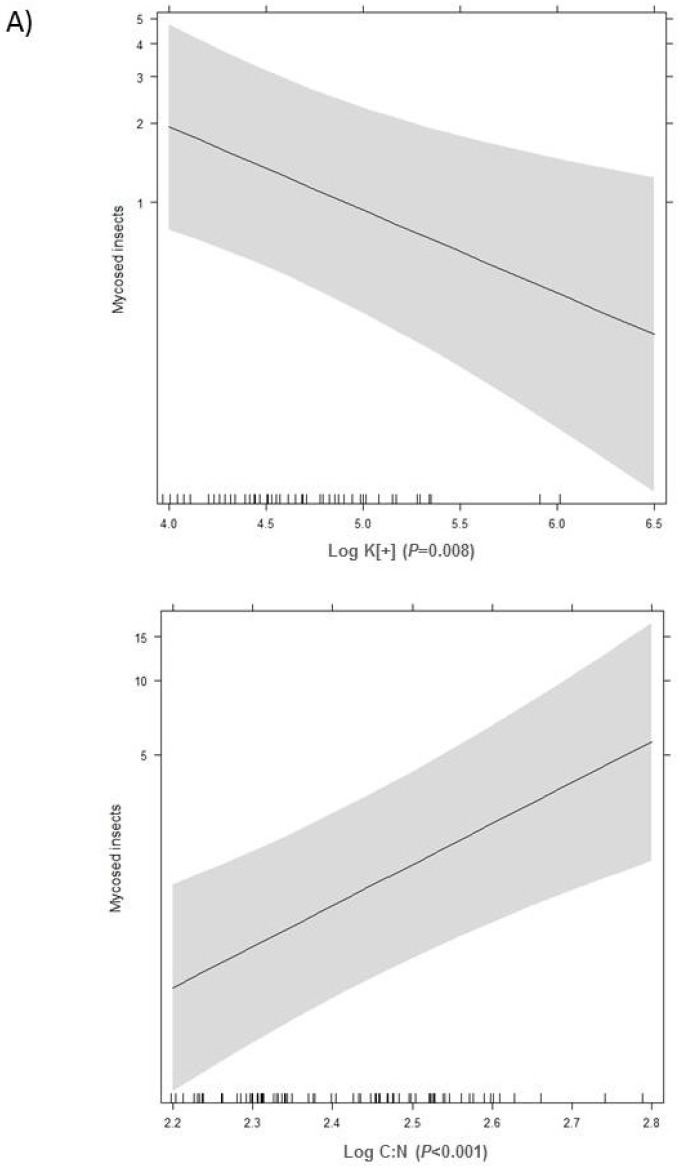

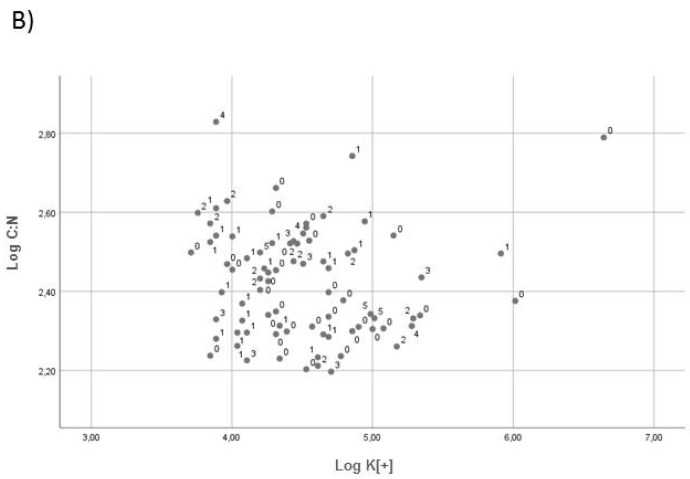
Variations in the number of insect larvae mycosed by *Fusarium oxysporum* with respect to the significant soil chemical variables. (**A**) The trend of larval mycoses with respect to the soil variables. (**B**) Scatter-plot of the number of larval mycoses by *Fusarium oxysporum* (mentioned as counts) at 80 soil sampling sites, with respect to the significant soil variables.

Previously, a generalized linear model (GLM) was used to relate the count data of *F. oxysporum* abundance with soil chemical properties, followed by the stepwise procedure for the deletion of non-significant variables. The only significant soil properties observed were the two log-transformed variables (i.e., log C:N and log K^+^). This analysis was followed by a GLMM with only log C:N and log K^+^ as the relevant soil properties, and the farm type as a random effect. For GLMM, the global significance of the model was *p* < 0.001 (Wald χ^2^ = 17.516, d.f. = 2), and the AIC value was 228.7. Higher log C:N significantly promoted while higher exchangeable log K^+^ significantly inhibited the abundance of *F. oxysporum* mycoses in insect larvae, respectively (Figure 1). The statistical values of the significance for the log-transformed variables were Wald χ^2^ = 15.468, d.f. = 1, and *p* < 0.001 for log C:N, and Wald χ^2^ = 6.976, d.f. = 1, and *p* = 0.008 for log K^+^. Other relevant values for these significant variables were estimate = 3.8238, standard error = 0.9722, and Z value = 3.933 for log C:N; and estimate = −0.7271, standard error = 0.2753, and Z-value = −2.641 for log K^+^.

## 3. Discussion

Biological communities in soils are likely to be the most complex. Microorganisms in the soils are extremely diverse, and they contribute to numerous ecosystem services that are critical to the sustainable functioning of both natural as well as managed ecosystems [17]. Agroecosystems, for example, constantly lose nutrients through leaching, run-off, denitrification, removal of crop harvest, and residues, and hence are dependent on continuous external inputs of nutrients. Such losses are likely to affect lower trophic levels, and ultimately influence different ecosystems services, such as pest suppression [18]. Soil microbes can affect crop yield, either (a) directly, e.g., as crop pathogens; or (b) indirectly, by participating in soil structure modification, carbon and nutrient cycles, and food web interactions. In either of these cases, soil microbes ultimately influence crop productivity [17,19,20]. To bridge the gaps between these phenomena, the current study focuses on the soil chemical properties with respect to the abundance of IPF *F. oxysporum*.

The most significant soil variable was the C:N (*p* < 0.001), which promoted the abundance of mycoses in insect larvae. Nitrogen is essential to plant growth and added as fertilizers in soils, if necessary. However, it was noticed that addition of the NPK fertilizers eventually reduces the density of entomopathogens—for example, nematodes [21]. Moreover, fertilizing soils tend to reduce the internal biological control within agroecosystems [18]. Higher organic matter, and hence, the higher organic carbon, increases the cation exchange capacity of the soils, which ultimately increases fungal conidia attachment [8]. Therefore, an increase in C and a decrease in N, which lead to a higher C:N, eventually facilitated the abundance of IPF *F. oxysporum* in our study.

Modeling has been an integral part in predicting phenomena in entomology. For example, it has been used previously to estimate flight phenology of world-famous insect pests, such as the European grapevine moth, or *Lobesia botrana* (Denis and Schiffermüller) (Lepidoptera: Tortricidae), in the Douro vineyards [13]. A generalized linear mixed model, with a previous GLM stepwise deletion of non-significant variables, provides a better outlook towards finding the variables that are significantly affecting the data. The stepwise GLM procedures allow the discarding of effects that do not differ significantly from zero. Further usage of a less complex model, such as GLMM, which is widely used in ecology [15], allowed improving the model and generalizing conclusions.

## 4. Materials and Methods

### 4.1. Sampling Site

Soils were initially collected in a previous study [1]. In the present study, the four farms, i.e., Aciprestes (41°12′25.2” N 7°25′55.2” W), Carvalhas (41°11′12.9” N 7°32′41.5” W), São Luiz (41°9′22′′ N 7°36′55′′ W), and Granja (41°15′18” N 7°28′34” W) were considered. These farms are located in the “Cima Cargo” region of the Douro vineyards of Portugal. The mean annual rainfall and temperature at the farms of São Luiz, Carvalhas, and Aciprestes ranges between 800–1000 mm and 14–16 °C, respectively. The Granja farm records 1000–1200 mm mean annual rainfall and temperatures ranging from 12–14 °C. Information on any chemical treatments of the soils is provided in the Supplementary Materials (see Table S1).

### 4.2. Fungal Isolation, Identification, and Screening

Soils were brought within the campus, and approximately one kg of those soils was air-dried and preserved for physicochemical analyses. For isolation of the entomopathogenic *F. oxysporum*, the remaining soil portions were equilibrated for moisture overnight, and then baited with eight late-instar larvae of *G*. *mellonella* and eight late-instar larvae of *Tenebrio molitor* Linnaeus (Coleoptera: Tenebrionidae) within 24 h, as described in a previous study [1]. In brief, two sets of four insect larvae of each bait insect were used. To reduce the tendency of silk web formation, the larvae of *G. mellonella* were given a heat shock in the water bath at 56 °C prior to baiting. Soils were kept at a temperature of 22 °C and a relative humidity of 85%, in the dark inside an environmental chamber (Panasonic MLR-352H-PE). Bowls were frequently agitated and inverted to maximize larval reach for fungal spores in soils. The total incubation period was three weeks. Insect cadavers were monitored every second day to retrieve any mycosed larvae, and to discard cadavers that were infected by entomopathogenic nematodes. Cadavers with a foul smell were also regularly discarded. These schedules were monitored rigorously. Insect cadavers that were suspected to be mycosed by the fungus were then washed for three minutes with 1% NaOCl, followed by three distinct washes with 100 mL of sterilized water. Subsequent culturing on potato dextrose agar was conducted until pure cultures were obtained. Insects were procured as described in another study [2]. *Fusarium oxysporum* have diverse ecological roles, and therefore, insect baiting seemed a better approach than soil suspension culture or a DNA-based approach for the accurate functional annotation of the obtained *F. oxysporum* isolate. Fungus was identified using morphological and molecular techniques, as described previously [1]. Infectivity of the isolated fungi were further confirmed by Koch’s postulates, as previously described [1,22,23]. Only the fungi that were found to be pathogenic after confirming Koch’s postulates were further considered in the study. A total of 80 samples were tested for the presence and abundance of entomopathogenic *F. oxysporum*.

### 4.3. Soil Analyses and Calculations

Soil pH was determined one hour after preparing a soil–water suspension. Organic matter content was determined using a total organic carbon analyzer (Primacs^SNC-100^, Skalar Analytical, Breda, The Netherlands). Total nitrogen was assessed by the Kjeldahl method, and the quantification was done using molecular absorption spectrophotometry [24]. The Egnér–Riehm method was used to extract P and K, and a spectrophotometer and a flame emission photometer (iCE™ 3300 AAS, Thermo Scientific^TM^, Breda, North Brabant, The Netherlands) was used for their respective determination. Exchangeable cations, or exchangeable bases, were measured by atomic absorption spectrophotometry, following the ammonium acetate extraction at a pH of 7.0 [25]. Titration method described in Thomas was used to determine exchangeable acidity [26]. Effective cation exchange capacity was calculated by summing exchangeable bases and exchangeable acidity. The degree of base saturation was measured by summing the exchangeable bases, dividing it by the ECEC, and then multiplying by 100.

### 4.4. Data Analyses

The abundance of the infected insects was analyzed using a generalized liner mixed model (GLMM), assuming a Poisson distribution for count data with a log link function. Model assumptions were inspected by visualizing residual plots. Soil properties were used as independent variables, herbicide application was considered as the fixed effect, and the farm type was considered as a random effect. The analysis started fitting the full model, which included all independent variables, followed by the stepwise procedure to remove non-significant variables [27]. The significance of the model was obtained using a Wald test, generated by the likelihood ratio tests of the full model with and without the explanatory variable. The analyses were performed in R (version 3.2.2) using the “MASS” package [28] and the “lme4” package [29].

## 5. Conclusions

Interactions between plants and microbes are quite complex, and it is necessary to move forward from a simplistic view of an individual plant–microbe interaction to all factors influencing agroecosystems. Soil, its microbes, and plants all work in coherence, and influence various exchanges contributing to plant health and productivity [30]. Soil provides fundamental ecosystem services, which include control of pests and diseases, nutrient cycling, and transformation of toxic materials and organic compounds. Microbes play a critical role in most of the soil processes. In this study, soil chemical properties affecting the presences of IPF *F. oxysporum* were investigated, and few significant findings could be made. Overall, it was noticed that entomopathogenic *F. oxysporum* is robust to most of the agricultural disturbances, although higher C:N and less exchangeable K^+^ might facilitate its natural abundance. This study suggests that IPF *F. oxysporum* can survive effectively in different soils, which further highlights its capabilities as an excellent soil saprophyte in the absence of host insects, as hinted previously [5]. This kind of approach can be extended to other beneficial soil microbes. Predicting soil microbial quality based on soil chemical properties could be a promising approach in the development of the methods for sustainable agriculture. Authors also suggest the use of GLMM in similar studies focusing on count data profiles, while accessing the factors affecting the abundance of the microbes of interest.

## Figures and Tables

**Table 1:** Soil physicochemical properties of the collected samples, and the numbers of entomopathogenic *Fusarium oxysporum* encountered.

Farm Type	Soil Type	Herbicide Usage	Rapid Texture	Collection Site	OM	P	K	Ca^2+^	Mg^2+^	K^+^	Na^+^	ECEC	N	TA	pH	C:N	DBS	*Fusarium oxysporum* Count
Carvalhas	Vineyards	0	Medium	nh1mCa1A	6.31	22	139	7.94	1.55	0.68	0.10	10.36	3.01	0.10	5.9	12.155174	99	1
Carvalhas	Vineyards	0	Medium	nh1mCa2C	4.57	6	90	4.27	1.20	0.33	0.03	5.83	2.45	0.00	6.3	10.819543	100	3
Carvalhas	Vineyards	1	Medium	hmCa3B	2.21	221	197	3.89	0.69	0.63	0.01	5.78	1.38	0.55	5.2	9.2963577	90	2
Carvalhas	Vineyards	1	Medium	hmCa4A	1.43	46	60	3.47	1.76	0.26	0.08	6.55	0.93	0.98	5.5	8.934769	85	1
Carvalhas	Vineyards	1	Medium	hmCa5A	1.39	45	56	4.94	1.17	0.19	0.18	6.71	0.90	0.23	5.8	8.9326187	97	1
Carvalhas	Vineyards	1	Gross	hgCa6B	4.15	96	84	1.09	0.37	0.26	0.03	4.15	2.21	2.40	4.4	10.895056	42	2
Carvalhas	Vineyards	1	Medium	hmCa7	1.24	91	92	4.10	1.07	0.28	0.03	6.22	0.89	0.75	5.3	8.0555353	88	0
Carvalhas	Vineyards	1	Medium	hmCa8	3.17	81	147	5.06	0.91	0.48	0.06	6.99	2.04	0.50	5.2	9.0237878	93	0
Carvalhas	Vineyards	1	Medium	hmCa9	1.76	106	118	3.33	0.67	0.40	0.02	5.72	1.22	1.30	4.9	8.3599869	77	0
Carvalhas	Vineyards	1	Medium	hmCa10	2.78	56	74	3.68	1.24	0.27	0.06	6.22	1.81	0.98	5.0	8.894316	84	0
Carvalhas	Vineyards	1	Medium	hmCa11A	2.57	70	108	3.50	0.80	0.41	0.10	6.36	1.69	1.55	4.6	8.8276961	76	1
Carvalhas	Vineyards	1	Medium	hmCa12A	2.74	64	104	6.24	1.49	0.36	0.03	8.70	1.79	0.58	5.3	8.8875571	93	1
Carvalhas	Hedgerows	0	Medium	nh2mCaM1B	5.71	14	86	8.17	1.43	0.37	0.07	10.25	2.90	0.00	6.4	11.438218	100	2
Carvalhas	Hedgerows	0	Medium	nh2mCaM2D	5.74	15	90	8.14	1.60	0.39	0.06	10.19	2.83	0.00	6.2	11.758777	100	4
São Luiz	Vineyards	1	Medium	hmSl11	4.38	174	82	2.41	1.87	0.30	0.10	4.93	2.22	0.25	5.5	11.44503	95	0
São Luiz	Vineyards	0	Medium	nh1mSl12	1.54	37	40	4.27	1.04	0.13	0.08	5.53	0.80	0.00	6.1	11.161607	100	0
São Luiz	Vineyards	1	Medium	hmSl21	6.38	34	72	6.09	1.71	0.25	0.05	8.09	2.96	0.00	6.2	12.492244	100	0
São Luiz	Vineyards	0	Medium	nh1mSl22A	3.41	28	68	3.78	0.83	0.21	0.03	4.85	1.85	0.00	6.3	10.680227	100	1
São Luiz	Vineyards	1	Medium	hmSl3A	2.09	56	50	8.10	1.65	0.14	0.08	9.96	1.21	0.00	6.8	9.9992	100	1
São Luiz	Vineyards	1	Medium	hmSl41A	1.26	26	58	3.46	1.09	0.14	0.06	4.74	0.79	0.00	5.9	9.2397671	100	1
São Luiz	Vineyards	0	Medium	nh1mSl42	2.93	43	108	3.81	1.17	0.32	0.06	5.35	1.82	0.00	6.1	9.3399121	100	0
São Luiz	Vineyards	1	Medium	hmSl51	6.84	45	74	1.34	0.69	0.24	0.06	2.74	2.98	0.40	5.6	13.314371	85	0
São Luiz	Vineyards	0	Medium	nh1mSl52	3.77	25	70	3.37	0.40	0.21	0.06	4.04	2.12	0.00	6.3	10.310496	100	0
São Luiz	Vineyards	1	Medium	hmSl61B	4.04	28	46	3.12	2.61	0.18	0.17	6.14	1.94	0.05	5.8	12.086662	99	2
São Luiz	Vineyards	0	Medium	nh1mSl62B	3.13	23	42	4.30	2.29	0.17	0.24	7.06	1.46	0.05	5.9	12.44421	99	2
São Luiz	Vineyards	1	Medium	hmSl71A	2.48	14	48	2.85	2.56	0.16	0.15	5.79	1.23	0.08	5.3	11.690122	99	1
São Luiz	Vineyards	0	Medium	nh1mSl72	2.07	6	52	2.45	2.56	0.15	0.13	5.36	1.11	0.08	5.5	10.809946	99	0
São Luiz	Vineyards	1	Medium	hmSl81A	1.59	38	76	2.58	1.36	0.19	0.06	4.18	1.01	0	6.4	9.1081822	100	1
São Luiz	Vineyards	0	Medium	nh1mSl82	1.90	53	108	1.76	0.85	0.23	0.12	2.96	1.1	0	6.0	9.9992	100	0
São Luiz	Vineyards	1	Medium	hmSl91	1.29	42	70	1.44	0.43	0.15	0.03	2.18	0.71	0.13	5.9	10.562535	94	0
São Luiz	Vineyards	0	Medium	nh1mSl92	1.71	51	54	1.86	0.43	0.15	0.05	2.48	0.93	0	6.2	10.64431	100	0
São Luiz	Vineyards	1	Medium	hmSl101	2.18	29	74	2.43	0.67	0.22	0.06	3.38	1.19	0	6.4	10.629402	100	0
São Luiz	Vineyards	0	Medium	nh1mSl102	4.02	20	92	3.76	0.77	0.25	0.03	4.82	1.93	0	6.3	12.081935	100	0
São Luiz	Vineyards	1	Medium	hmSl111B	2.31	10	66	7.18	1.39	0.15	0.07	8.79	1.29	0	6.6	10.386766	100	2
São Luiz	Vineyards	0	Medium	nh1mSl112A	2.88	45	60	8.14	1.23	0.16	0.08	9.61	1.52	0	6.7	10.985963	100	1
São Luiz	Vineyards	0	Medium	nh1mSl112	4.22	7	70	11.23	1.36	0.16	0.15	12.91	2.61	0	6.8	9.3862222	100	0
São Luiz	Vineyards	1	Medium	hmSl131B	2.79	50	104	2.19	0.69	0.28	0.05	3.22	1.31	0	6.1	12.334891	100	2
São Luiz	Vineyards	0	Medium	nh1mSl132	2.13	49	94	3.33	0.59	0.26	0.03	4.21	1.07	0	6.3	11.531788	100	0
São Luiz	Vineyards	1	Medium	hmSl141A	2.98	59	129	1.78	0.24	0.32	0.03	2.37	1.54	0	5.6	11.232868	100	1
São Luiz	Vineyards	0	Medium	nh1mSl142A	3.05	52	368	2.42	0.59	0.51	0.06	3.57	1.59	0	6.2	11.131185	100	1
São Luiz	Vineyards	1	Gross	hgSl151A	10.96	79	127	6.98	2.45	0.47	0.08	10.93	4.38	0.95	6.1	14.519386	91	1
São Luiz	Vineyards	0	Medium	nh1mSl152	1.57	8	74	1.71	0.77	0.20	0.07	2.76	0.96	0	5.6	9.4784083	100	0
São Luiz	Vineyards	1	Gross	hgSl241	16.37	152	767	6.12	3.73	0.88	0.13	10.87	6.22	0	6.3	15.262445	100	0
São Luiz	Vineyards	0	Medium	nh1mSl242	5.16	51	171	5.39	1.36	0.43	0.03	7.22	2.56	0	6.3	11.694377	100	0
São Luiz	Vineyards	0	Medium	nh1mSl26A	2.17	64	54	7.52	2.45	0.12	0.10	10.54	1.08	0.35	7.0	11.665733	97	1
São Luiz	Hedgerows	0	Gross	nh2gSlM1A	9.02	4	48	3.12	1.36	0.14	0.10	4.72	4.15	0	5.4	12.601401	100	1
São Luiz	Hedgerows	0	Gross	nh2gSlM2D	12.84	5	48	3.89	1.49	0.16	0.86	6.40	4.68	0	6.7	15.91753	100	4
São Luiz	Hedgerows	0	Gross	nh2gSlM3B	8.55	2	52	3.18	1.39	0.29	0.14	5.50	3.86	0.50	6.0	12.848713	91	2
São Luiz	Hedgerows	0	Gross	nh2gSlM4A	6.79	1	46	1.82	0.83	0.16	0.08	3.27	3.43	0.38	5.9	11.485962	89	1
Granja	Vineyards	1	Medium	hmGr21	1.41	48	408	3.5	1.0	0.9	0.4	5.87	0.84	0.09	5.8	9.7611238	98	0
Granja	Vineyards	0	Medium	nh1mGr22E	1.10	24	145	3.2	0.8	0.4	0.3	5.25	0.68	0.585	5.1	9.4110118	89	5
Granja	Vineyards	1	Medium	hmGr31	1.19	6	133	2.4	1.0	0.3	0.3	4.30	0.76	0.315	4.6	9.0782211	93	0
Granja	Vineyards	0	Medium	nh1mGr32C	0.98	11	60	3.6	0.8	0.2	0.3	5.28	0.69	0.338	4.6	8.2602087	94	3
Granja	Vineyards	1	Medium	hmGr41	2.07	22	207	1.7	0.7	0.7	0.4	4.29	1.28	0.72	4.7	9.37425	83	0
Granja	Vineyards	0	Medium	nh1mGr42C	2.57	32	209	1.7	0.7	0.7	0.3	3.90	1.43	0.563	5.0	10.418747	86	3
Granja	Vineyards	1	Medium	hmGr51B	1.05	30	175	1.6	0.5	0.4	0.3	3.48	0.71	0.63	5.0	8.590862	82	2
Granja	Vineyards	0	Medium	nh1mGr52D	1.40	25	195	1.4	0.4	0.6	0.3	3.32	0.89	0.608	5.0	9.1003955	82	4
Granja	Vineyards	1	Medium	hmGr61	1.52	17	120	1.9	0.7	0.3	0.4	4.22	0.90	0.945	4.4	9.7769956	78	0
Granja	Vineyards	0	Medium	nh1mGr62E	1.38	11	149	1.4	0.4	0.4	0.3	3.83	0.86	1.35	4.4	9.3015814	65	5
Granja	Vineyards	1	Medium	hmGr71A	0.78	10	100	2.2	0.9	0.3	0.3	4.31	0.54	0.675	4.7	8.3326667	84	1
Granja	Vineyards	0	Medium	nh1mGr72C	0.55	7	110	1.3	0.5	0.3	0.3	3.18	0.40	0.72	4.8	7.99936	77	3
Aciprestes	Vineyards	1	Medium	hmAc11	1.35	100	100	4.19	0.85	0.16	0.13	5.33	0.96	0	7.2	8.1347658	100	2
Aciprestes	Vineyards	0	Medium	nh1mAc12	2.08	96	108	4.48	1.36	0.21	0.03	6.08	1.13	0	7.0	10.68941	100	1
Aciprestes	Vineyards	1	Medium	hmAc21	2.32	90	84	6.11	1.07	0.15	0.06	7.38	1.17	0	7.5	11.511899	100	3
Aciprestes	Vineyards	0	Medium	nh1mAc22	3.04	106	72	6.54	1.71	0.13	0.15	8.53	1.54	0	7.3	11.453629	100	1
Aciprestes	Vineyards	1	Medium	hmAc3	2.55	214	104	4.67	0.96	0.20	0.04	5.87	1.36	0	7.2	10.888835	100	1
Aciprestes	Vineyards	1	Medium	hmAc41	1.31	66	66	3.90	1.20	0.11	0.09	5.31	0.68	0	7.3	11.160872	100	5
Aciprestes	Vineyards	0	Medium	nh1mAc42	1.24	83	46	6.4	1.1	0.1	0.4	7.96	0.86	0	6.9	8.3714233	100	0
Aciprestes	Vineyards	1	Medium	hmAc51	0.51	17	127	3.94	1.55	0.09	0.11	5.68	0.33	0	7.3	8.9689794	100	0
Aciprestes	Vineyards	0	Medium	nh1mAc52	0.88	28	48	4.8	1.3	0.2	0.3	6.67	0.55	0	6.7	9.2719855	100	3
Aciprestes	Vineyards	1	Medium	hmAc61	0.99	91	76	5.84	1.44	0.12	0.09	7.49	0.69	0	7.0	8.3036835	100	0
Aciprestes	Vineyards	0	Medium	nh1mAc61	2.16	100	58	6.9	1.9	0.2	0.5	9.49	1.29	0	6.7	9.6891473	100	1
Aciprestes	Vineyards	1	Medium	hmAc71	1.46	59	96	4.78	1.44	0.11	0.06	6.40	0.93	0	7.2	9.0852946	100	0
Aciprestes	Vineyards	0	Medium	nh1mAc72	0.62	34	48	4.4	1.2	0.1	0.1	5.92	0.41	0	6.9	8.7797854	100	1
Aciprestes	Vineyards	1	Medium	hmAc81	1.98	206	159	6.18	1.33	0.13	0.07	7.71	1.27	0	7.3	9.0386469	100	0
Aciprestes	Vineyards	0	Medium	nh1mAc82	1.19	73	80	7.9	1.5	0.2	0.4	9.95	0.77	0	6.9	8.9603221	100	0
Aciprestes	Hedgerows	0	Medium	nh2mAcM1	2.62	12	92	4.08	1.44	0.18	0.06	5.77	1.27	0	6.8	11.9518	100	0
Aciprestes	Hedgerows	0	Gross	nh2gAcM2	1.61	4	66	3.07	1.87	0.14	0.04	5.12	0.93	0	6.7	10.063711	100	2
Aciprestes	Hedgerows	0	Medium	nh2mAcM3	1.75	20	123	3.39	1.28	0.23	0.03	4.93	0.91	0	6.7	11.130978	100	2
Aciprestes	Hedgerows	0	Medium	nh2mAcM4	0.56	4	56	3.30	1.81	0.10	0.03	5.24	0.38	0	6.6	8.6045747	100	1

Note: herbicide applied (1) and no herbicide applied (0). Other representations are organic matter % (OM), available phosphorous mg/kg (P), available potassium mg/kg (K), exchangeable calcium ions cmol/kg (Ca^2+^), exchangeable magnesium ions cmol/kg (Mg^2+^), exchangeable potassium ions cmol/kg (K^+^), exchangeable sodium ions cmol/kg (Na^+^), effective cation exchange capacity cmol/kg (ECEC), total nitrogen g/kg (N), total acidity (TA), and degree of base saturation % (DBS).

## Supplementary Materials

The following are available online at http://www.mdpi.com/2076-0817/7/4/89/s1, Table S1: Chemicals applied in the farms of the Douro wine region.
